# Hepatic epithelioid hemangioendothelioma: A comparison of Western and Chinese methods with respect to diagnosis, treatment and outcome

**DOI:** 10.3892/ol.2014.1847

**Published:** 2014-02-03

**Authors:** XIN YAN ZHAO, MOHMEDMOHSIN ISMAIL AHMED RAKHDA, SOHAIL HABIB, ALI BIHI, ABDULLAH MUHAMMAD, TAI LING WANG, JI-DONG JIA

**Affiliations:** 1 Liver Research Center, Beijing Friendship Hospital, Capital Medical University, Beijing 100050, P.R. China; 2 International School of Capital Medical University, Beijing 100069, P.R. China; 3 Department of Pathology, China-Japan Friendship Hospital, Beijing 100029, P.R. China

**Keywords:** liver resection, liver transplantation, natural history, prognostic factor, hepatic epithelioid hemangioendothelioma

## Abstract

Hepatic epithelioid hemangioendothelioma (HEHE) is a rare tumor of vascular origin. Whether HEHE in Chinese patients exhibits similar characteristics compared with Western patients is not well known. The aim of the present study was to summarize the characteristics of HEHE in Chinese patients and identify its prognostic factors. In total, six patients diagnosed with HEHE at the Beijing Friendship Hospital between 2000 and 2012 were combined with 44 previously reported cases in China, retrieved from the literature between 1989 and mid-2012. These 50 cases from China were compared with 402 patients from Western populations. Prognostic factors were identified by the χ^2^ test and Cox regression analysis. The male to female ratio of the Chinese patients was 1:2.1 with the mean age of 44.2 years (range, 22–86 years). The percentage of asymptomatic Chinese patients was significantly higher than in the Western patients (40.0 vs. 24.8%; P=0.026), and that of extrahepatic metastasis (16.0 vs. 36.6%; P=0.005) was significantly lower in Chinese patients. On imaging study, capsular retraction (59.5%) and calcification (26.0%), as well as positivity of CD34 (93.5%) and CD31 (80.6%), were more frequently found in the Chinese patients. Management for the Chinese patients included liver resection (LRx; 45.7%), liver transplantation (LTx; 5.7%), trans-catheter arterial chemoembolization (14.3%) and palliative treatment (34.3%). Chinese patients with larger-sized tumor nodules [relative risk (RR), 1.58; 95% confidence interval (CI), 1.032–2.422; P=0.035) and diffuse type (RR, 12.17; 95% CI, 1.595–92.979; P=0.016) exhibited unfavorable outcomes. In contrast to Western patients with HEHE, a larger number of Chinese patients were asymptomatic with less extrahepatic metastasis. In China, LRx is widely adopted rather than LTx. Chinese patients with large tumor size or diffuse type may encounter a poorer prognosis.

## Introduction

Epithelioid hemangioendothelioma (EHE) is a rare tumor of vascular origin (with an incidence of less than one in 1,000,000 worldwide) with low to moderate grade malignant properties. EHE was initially described in 1982 by Weiss and Enzinger (1). Hepatic EHE (HEHE) mainly affects middle-aged females with a variable and unpredictable clinical course. The survival time ranges between 5 and 28 years to a rapidly progressing disease with a fatal outcome (2,3). Thus far, no definitive factors for the development of HEHE have been identified; although, oral contraceptive pills, exposure to vinyl chloride, liver trauma and viral hepatitis may be implicated. In addition, the prognostic factors have not been clearly defined (4).

Relatively fewer cases of HEHE (mainly in Western countries) have been reported worldwide (4,5). However, HEHE in China has been reported in small case series. Therefore, whether HEHE in the Chinese population exhibits different characteristics than that of Western patients has not been well summarized or compared, and this may be due to the rarity of the disease and the small number of previously reported cases.

The present study compared 50 Chinese patients with HEHE to 402 Western patients with HEHE [summarized by Mehrabi *et al* (5)], in order to describe the characteristics and identify the prognostic factors of HEHE in Chinese patients.

## Materials and methods

The data of six patients with HEHE that received consultation at the Beijing Friendship Hospital between 2000 and 2012 were collected. The patients’ medical records were retrospectively reviewed with regard to clinical manifestations, laboratory observations, imaging with pathological features, treatment and clinical outcome. Pathological diagnosis of HEHE was reconfirmed by a pathologist from the China-Japan Friendship Hospital (Beijing, China) on the basis of the presence of CD34- or CD31-positive epithelioid or dendritic endothelial cells infiltrating the hepatic sinusoids. The study protocol was approved by the Research Ethics Committee of the Beijing Friendship Hospital, Capital Medical University (Beijing, China).

Furthermore, literature searches were conducted via PubMed, Medline and the China National Knowledge Infrastructure (CNKI) using the terms ‘hepatic epithelioid hemangioendothelioma’ or ‘epithelioid hemangioendothelioma of the liver’ between the years of 1989 and mid-2012. Chinese patients with HEHE whose cases were published in English or at least provided an abstract in English, were included. The six patients diagnosed at the Beijing Friendship Hospital were evaluated with the 44 published Chinese cases (a total of 50 cases) and compared with 402 Western patients with HEHE. The patient data were obtained from an extensive review by Mehrabi *et al* (5)*,* based on the published literature between 1984 and 2005. Patient information, such as age, gender, clinical manifestation, diagnostic tools, histopathological aspects, treatments and outcomes, were documented in the review by Mehrabi *et al*. Therefore, this information from the Western HEHE patients could be compared with that of the Chinese patients.

Among the Chinese patients with HEHE, the final outcome was available in 25 patients (the remaining patients succumbed during follow-up), those who remained alive (n=16) were compared with those who had succumbed to the disease (n=9). This was in order to identify risk factors of poor prognosis, and the χ^2^ test and Cox regression analysis were used.

### Statistical analyses

Frequency distribution and differences between the 50 Chinese and 402 Western cases, including the 25 Chinese patients who survived or who had succumbed to the disease, were determined by the χ^2^ test for qualitative results and Student’s t-test for quantitative results. Log-rank survival analysis was performed using Kaplan-Meier methods to test differences in survival (in months) between Chinese patients who underwent surgery [liver transplantation (LTx) and liver resection (LRx)], trans-catheter arterial chemoembolization (TACE) and palliative treatment. P<0.05 was considered to indicate a statistically significant difference. A backward stepwise Cox regression model was used to identify risk factors for poor prognosis. The following criteria were used for inclusion and exclusion: Inclusion, P<0.05; and exclusion, P>0.10.

## Results

### Summary of the six Chinese patients diagnosed with HEHE and the follow-up

**Figure 1:**
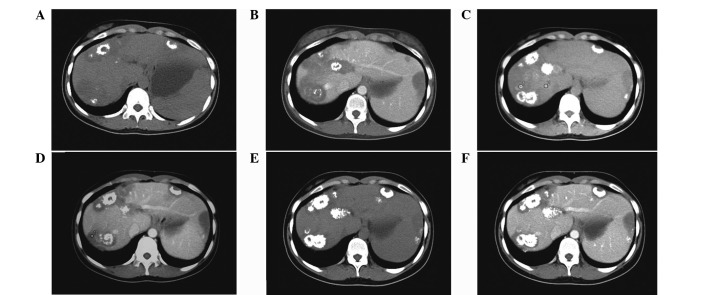
Three-year follow up CT images of one patient (no. 4) who did not receive any specific therapy. (A and B) First plain CT image showed multiple hypoattenuating coalescent nodules in the peripheral region of the liver with capsular retraction and mild calcification. (C and D) Second CT (one-year later) showed no change in tumor size, but an increase in calcification. (E and F) Third CT examination (two-years later) reconfirmed the regression of the tumor with the augmentation of calcification replacing the previous tumor cells. CT, computed tomogrphy.

Over the past 11 years, six patients with pathologically confirmed HEHE were identified. The demographic and clinical characteristics, including treatment and outcome of these patients, are summarized in Table I. The biochemical parameters of the six patients included mildly elevated alkaline phosphatase (ALP), γ glutamyl transpeptidase (GGT; 3/6), alanine transaminase (ALT) and aspartate transaminase (AST; 1/6) levels. The viral markers for hepatitis B virus (HBV) and hepatitis C virus were all negative. Calcification was found in four patients and capsular retraction in three patients (Fig. 1). A total of two out of the six patients exhibited positive ‘target’ and ‘halo’ signs.

**Figure 2:**
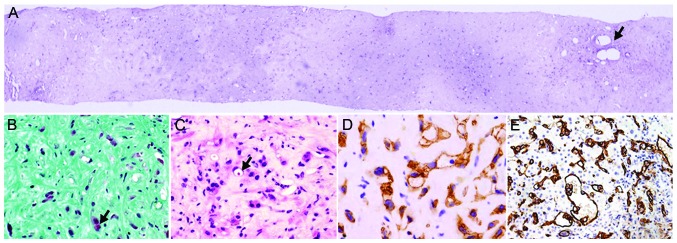
Histopathology of hepatic epithelioid hemangioendothelioma. (A) Percutaneous liver biopsy of one of the tumor nodules showed liver parenchyma replacement by tumor cells with mucoid stroma and sparing of portal tract, as indicated by the arrow. Periodic acid Schiff staining (magnification, ×10). (B) Scanty tumor cells, in red and indicated by the arrow, embedded in mucopolysaccharide-rich stroma and excessive extracellular matrix following Masson staining. Masson’s Trichrome stain (magnification, ×40). (C) Adjacent to the center of the tumor, increased epithelioid (intracytoplasmic lumina containing erythrocytes, as indicated by the arrow) or dendritic tumor cells with stroma were observed. Hematoxylin and eosin staining (magnification, ×40). Positive CD34 epithelioid cells (D) scattered in the tumor stroma (CD34 staining; magnification, ×80) and (E) infiltrating the sinusoids, creating the potential for tumor spread (CD31 staining; magnification, ×40).

Diagnosis of HEHE was established in all six Chinese patients by core liver biopsy, according to the presence of CD34/CD31-bearing epithelioid or dendritic endothelial cells (Fig. 2). All six patients received supportive therapy and one patient (no. 6) was on the waiting list for LTx. A total of three out of the six patients survived, with the survival period ranging between 16 and 36 months. One patient (no. 4; without treatment) exhibited an evident increase in the quantity of calcification, but the tumor remained stable even showing self-regression over three years (Fig. 1). In addition, two patients succumbed to liver failure due to the progression of the tumor and the remaining patient did not return for follow-up. No identifiable underlying risk factors were identified.

### Summary and analyses of 50 Chinese patients with HEHE, and their comparison with 402 HEHE patients from Western populations

A total of 11 studies were retrieved from PubMed, but two were excluded (mainly for not focusing on HEHE). Therefore, nine studies (38 patients), including patients with primary HEHE from mainland China (6–10), Hong Kong (11) and Taiwan (12–14), were subsequently included in the study. In addition, a Chinese case report that included eight patients was identified on the CNKI database and was also included (15). Of these eight patients, two were diagnosed with HEHE co-existing with hepatocellular carcinoma (HCC) and, consequently, were excluded from the study. Thus, a total of 44 (previously published) Chinese patients with HEHE and six Chinese patients diagnosed with HEHE at the Beijing Friendship Hospital were analyzed as a whole in the current study. Specimens obtained for pathology were confirmed through liver biopsy (62%), wedge LRx (34%) and LTx (4%).

### Demographic and clinical manifestation

The male to female ratio of the 50 Chinese patients was 1:2.1 and the mean age was 44.2 years (ranging between 22 and 86 years; Table II). The presenting symptoms of HEHE in the 50 Chinese patients were non-specific and 40.0% of patients were asymptomatic, which is significantly higher than in the 402 Western cases (40.0 vs. 24.8%; P=0.026). Among these 50 patients, the serum tumor markers, including CA-125 (4%), CEA (2%) and CA-199 (2%), were marginally elevated with the exception of α-fetoprotein. The surface antigen of the HBV, HBsAg, was positive in 12.2% of patients.

### Pathological features, extrahepatic metastasis, treatment and survival time

In terms of pathology, three different patterns of HEHE were identified, solitary (14.0%), multiple (78.0%) and diffuse (8.0%) types. The progression of multiple to diffuse type was recorded in three patients. The characteristic feature of HEHE was zonal distribution. In the midzone, scanty tumor cells were distributed within mucopolysaccharide stroma or fibrotic tissue (Fig. 2A and B). The tumor cells (epithelioid or dendritic) were present at the periphery, invading into sinusoids with atrophy or disappearance of the adjacent hepatic plate was observed (Fig. 2C–E). In addition, tumor cells were observed to infiltrate the branches of the portal vein and/or hepatic vein (26.9%). Positivity of CD34 (93.5%) and CD31 (80.6%) were comparable between the 50 Chinese and 402 Western patients.

Metastasis outside of the liver was significantly lower than in the Western population (16 vs. 36.6%; P=0.005); although, the lung (3/8) was the most common site of metastasis, followed by bone (2/8), spleen (1/8) and abdominal wall (1/8). One patient exhibited multiple site metastasis, including the lung, pericardium, spleen and bone.

The management for the 50 Chinese patients included LRx (45.7%), LTx (5.7%) and TACE (14.3%). Patients without specific treatment were 34.3% and final outcome was available for 25 Chinese patients. Regardless of treatment, 16 patients survived with the mean survival time of 31 months (maximal survival time was approximately eight years). In total, nine patients succumbed to the disease, with a mean survival time of 27 months. Although, Kaplan-Meier analysis showed a trend of improvement in survival time with liver surgery compared with TACE or palliative therapy. No significant difference was identified in overall survival among these treatment options (P=0.741). A total of two out of the seven (28.6%) patients who survived following LRx exhibited recurrence of the disease.

### Identification of factors in Chinese patients with HEHE as predictors for clinical outcomes

By χ^2^ analysis (Table III) the percentage of asymptomatic patients was significantly higher in patients who had survived (56.3%) compared with the group of patients who had succumbed to their diseases (11.1%) (P=0.027). Although tumor markers were non-specific for HEHE, a higher proportion of abnormality of tumor markers (33.3%) was identified in the patients who had succumbed to their diseases compared with the patients who had survived (6.2%) (P=0.076). No significant difference was identified in extrahepatic metastasis between the two groups.

By a backward stepwise COX regression model, the relative risk (RR) in patients with diffuse type of the disease was 12.17 [95% confidence interval (CI), 1.595–92.979; P=0.016] and tumor size was 1.58 (95% CI, 1.032–2.422; P=0.035). Again, presence of extrahepatic metastasis was not an independent risk factor for poor prognosis (RR, 0.025; 95% CI, 0–13; P=0.247).

## Discussion

HEHE is a rare malignant tumor of vascular origin and an intermediate between hemangioma and angiosarcoma in nature. EHE was first reported in liver tissue by Ishak *et al* in 1984 (16). Its vascular origin is supported by positive staining for factor VIII-related antigen. The characteristics of HEHE in the Chinese population are not well known. Therefore, the present study reported six patients diagnosed in the Beijing Friendship Hospital and retrospectively reviewed 44 patients from the previously published literature, not only from mainland China but also from Taiwan and Hong Kong. The current study presents a large-scale summary of HEHE in China.

The present study found that HEHE mainly affects Chinese adults during their mid-forties, with a higher prevalence in females; the male to female ratio of the Chinese patients was 1:2.1, consistent with the 402 previously published cases. Notably, 40% of the 50 Chinese patients were diagnosed incidentally, which is a significantly higher proportion than that in the previously published studies. At present, increasing awareness of routine checkup in the Chinese population contributes to the early detection of this rare disease even at the asymptomatic stage. Mildly elevated ALP and GGT are the most common types of abnormal liver function tests (LFTs). However, LFTs revealed a significantly lower incidence of abnormality than in the 402 Western cases (40 vs. 80%) which may be explained by early detection of HEHE. Of note, LFTs may be within the normal range in the early stage of the disease, however, with progression of the disease, HEHE may involve more liver parenchyma and result in elevated liver enzymes in the later stages of the disease.

Patients who initially exhibit a solitary lesion may progress to exhibit multiple separate lesions, followed by multiple coalescing lesions, which eventually progress to diffuse pattern. The present study observed this progression in three patients. During the natural development of this disease, the patient exhibits an increased number of symptoms, signs and the involvement of abnormal liver enzymes. The final clinical outcomes include liver failure caused by loss of parenchyma or portal hypertension (tumor cells invading and occluding presinusoidal and postsinusoidal space). On the other hand, multiple lesions may undergo self-remission manifested by progressive calcification, even without specific therapy as shown in case no. 4 (Fig. 1).

Currently, LTx is an optimal treatment for unresectable tumors, even in patients who exhibit extrahepatic involvement with three- and five-year survival rates of ~80 and 70%, respectively (17–19). However, in China, few HEHE patients have been previously treated with LTx. LRx remains the most common choice for disease management where possible (20). Extrahepatic spread at the time of LRx does not correlate with survival and has not been considered a contraindication to surgery. Traditional chemotherapy does not appear to be an acceptable treatment option for this disease due to the insensitivity of the tumor cells to chemotherapeutic intervention. However, it has been previously reported that antiangiogenesis-based chemotherapy, such as thalidomide (21–23), may effectively prevent the progression of the disease and improve the clinical outcome.

**Figure 3:**
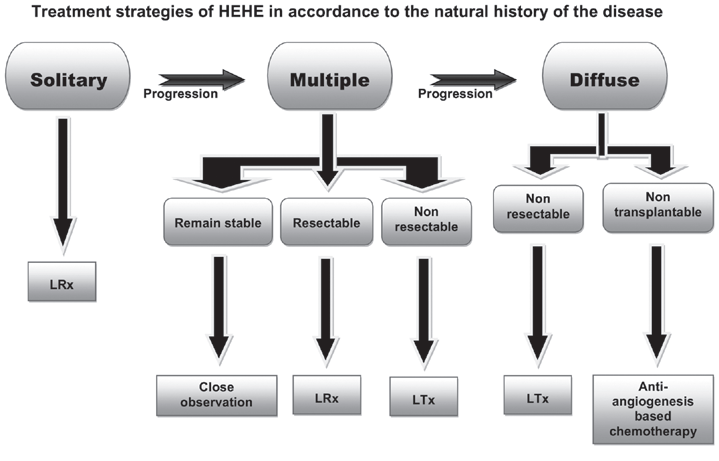
Treatment strategies of HEHE. Patients with a solitary pattern must undergo liver resection. In addition, patients with multiple nodules who remain stable and perform continuous follow-up with computed tomography or ulrasound examination (every 3–6 months) only require close monitoring. In case of any increase in size or number of nodules which are resectable, LRx is the optimum treatment, whereas, non-resectable multiple nodules must be treated by LTx. For patients with a diffuse pattern, LTx is the only treatment option. If there is no chance of LTx for any reason, antiangiogenesis-based chemotherapy is the preferred treatment option. HEHE, hepatic epithelioid hemangioendothelioma; LRx. liver resection; LTx, liver transplantastion.

Occasionally, it is difficult to select the most appropriate treatment (LRx, LTx or chemotherapy), individually, due to one or more of the following reasons. Firstly, unpredictable clinical course ranging between complete remission and rupture of tumor with a fatal outcome. Secondly, lack of first grade evidence from well controlled-clinical trials due to the rarity of the disease. Thirdly, certain insufficient treatments accelerate the process of the disease leading to an unfavorable outcome, such as insufficient LRx. Finally, unlike other tumors, such as HCC, extrahepatic metastasis is not an absolute contraindication of liver surgery. The current study proposes a treatment strategy based on the course of HEHE, which complies with the principle of monitoring closely and adapting properly, a strategy to fit each individual’s demand to optimize the treatment regimen for the most favorable outcome (Fig. 3).

Nevertheless, even without any treatment, 40% of patient conditions remain stable for a long period of time. Therefore, identifying risk factors which predict clinical course is extremely important to determine treatment-related decisions for the patients. In the current study, Chinese patients who had survived were compared with patients who had succumbed to their diseases. The results showed that patients who exhibited systemic symptoms, such as larger tumor size and diffuse type, carried an unfavorable outcome, similar to that in two previous studies by Grozt *et al* (24) and Wang *et al* (25).

Notably, unlike Western cases, a number of HEHE patients in China are asymptomatic with normal liver enzymes and exhibit less extrahepatic metastasis for a considerable part of the disease course. Furthermore, LRx rather than LTx is a more common treatment option in China. Finally, treatment of HEHE must be in accordance with the natural history of the disease and patients with individualized treatment may exhibit an improved clinical outcome.

## Figures and Tables

**Table I:** General condition, clinical manifestation, diagnosis, treatment and outcome of six patients with HEHE.

Patient	Gender/age, years	Presenting symptoms	Physical examination observations	Tumor number and location	Maximum size, cm	Treatment	Outcome (months)
1	F/49	RUQ pain	Pain induced by percussion on liver region	Multiple, the two lobes	4.1×2.7	None	Loss of follow-up
2	F/35	RUQ pain	Pain induced by percussion on liver region	Multiple, the two lobes	4.0×3.5	TCM	Survived (16)
3	F/56	RUQ discomfort and fatigue	Dark facial appearance and palmar erythema	Multiple, the two lobes	7.0×4.9	None	Succumbed to disease (10)
4	F/22	None	None	Multiple, the two lobes	3.6×3.1	None	Survived (17)
5	M/45	Abdominal distention weight loss	Hepatomegaly, shifting dullness and mild edema	Multiple, the two lobes	5.0×4.5	TCM	Succumbed to disease (79)
6	M/61	None	Hepatomegaly	Diffuse, the two lobes	3.0×2.3	None	Survived (36)

HEHE, hepatic epithelioid hemangioendothelioma; F, female; M, Male; RUQ, right upper quadrant; TCM, traditional Chinese medicine.

**Table II:** Comparison between Chinese and Western patients with HEHE.

Variables	Chinese patients (n=50)	Western patients (n=434)	P-value
Gender, M, % (M:F)	32 (16:34)	42.4 (154:209)	0.160
Mean age, years (range)	44.2 (22–86)	41.7 (3–86)	NA
Presenting symptoms, % (n)			
RUQ pain	32.0 (16/50)	48.6 (143/294)	0.029
Asymptomatic	40.0 (20/50)	24.8 (73/294)	0.026
Weight loss	10.0 (5/50)	15.6 (46/294)	0.299
Anorexia	6.0 (3/50)	8.2 (24/294)	0.599
Abdominal discomfort	26.0 (13/50)	5.4 (16/294)	<0.001
Positive physical signs, % (n)			
Hepatomegaly	18.0 (9/50)	20.4 (60/294)	0.694
Shifting dullness	8.0 (4/50)	6.8 (20/294)	0.759
Jaundice	6.0 (3/50)	6.5 (19/294)	0.902
Splenomegaly	8.0 (4/50)	2.4 (7/294)	0.037
Abdominal tenderness	4.0 (2/50)	1.0 (3/294)	0.104
Elevated ALP	27.5 (11/40)	68.6 (127/185)	<0.001
Elevated GGT	17.5 (7/40)	45.4 (84/185)	0.001
Elevated ALT	12.5 (5/40)	28.6 (53/185)	0.034
Elevated AST	17.5 (7/40)	23.2 (43/185)	0.428
Elevated Bilirubin	7.5 (3/40)	19.5 (36/185)	0.07
Imaging features, % (n)			
Low consistency	95.7 (44/46)	98.1 (102/104)	0.395
Location of the tumor, % (n)			
Two lobes	55.3 (21/38)	84.6 (259/306)	<0.001
Left lobe	5.3 (2/38)	2.0 (6/306)	0.203
Right lobe	39.5 (15/38)	13.4 (41/306)	<0.001
Capsular retraction	59.5 (22/37)	10.6 (15/142)	<0.001
Halo sign	37.5 (6/16)	10.4 (5/48)	0.005
Calcification	26.0 (13/50)	12.7 (18/142)	0.028
Type of involvement, % (n)			
Multiple focal including diffuse type	86.0 (43/50)	87.3 (267/306)	0.806
Unifocal	14.0 (7/50)	12.7 (39/306)	0.806
Extra hepatic metastasis	16.0 (8/50)	36.6 (90/246)	0.005
Immunohistopathological markers, % (n)			
CD34	93.5 (43/46)	94.5 (129/137)	0.799
CD31	80.6 (25/31)	86.1 (118/137)	0.438

HEHE, hepatic epithelioid hemangioendothelioma; RUQ right upper quadrant pain; ALP, alkaline phosphatase; GGT, γ glutamyl transpeptidase; ALT, alanine transaminase; AST, aspartate transaminase; NA, not applicable.

**Table III:** Comparison between Chinese patients who survived and succumbed to the disease in terms of demographic, clinical, laboratory and radiological parameters.

Parameters	Survived (n=16)	Succumbed (n=9)	P-value
Gender, F, % (F:M)	81.2 (13:3)	66.7 (6:3)	0.412
Age, years (mean ± SD)	43.1±12.7	46.8±19.5	0.568
Asymptomatic, % (n)	56.3 (9/16)	11.1 (1/9)	0.027
Tumor markers (CA199 and CEA), % (n)	6.2 (1/16)	33.3 (3/9)	0.076
Abnormality of LFTs, % (n)	31.2 (5/16)	55.6 (5/9)	0.234
HBsAg, % (n)	12.5 (2/16)	33.3 (3/9)	0.211
Tumor size, cm (mean ± SD)	4.4±1.6	5.2±2.2	0.342
Target sign, % (n)	36.4 (4/11)	33.3 (2/6)	0.901
Capsular retraction, % (n)	54.5 (6/11)	66.7 (4/6)	0.627
Calcification, % (n)	18.8 (3/16)	33.3 (3/6)	0.412
Multiple and diffuse type: solitary type, % (ratio)	87.5 (7:1)	88.9 (8:1)	0.918
Extrahepatic metastasis, % (n)	18.8 (3/16)	22.2 (2/9)	0.835
Life period, months (mean ± SD)	31±29.3	27±26.6	0.726

F, female; M, male; LFTs, liver function test; HBsAg, hepatitis B antigen; SD, standard deviation.

## Abbreviations

HEHE: hepatic epithelioid hemangioendothelioma
EHE: epithelioid hemangioendothelioma
LTx: liver transplantation
CNKI: China national knowledge infrastructure
RR: relative risk
ALP: alkaline phosphatase
GGT: γ-glutamyl transferase
TCM: traditional Chinese medicine
HCC: hepatocellular carcinoma
RUQ: right upper quadrant
ALT: alanine transaminase
LFTs: liver function tests
LRx: liver resection
